# A New Antagonizing Instrument for Double Sets of Artificial Teeth

**Published:** 1848-01

**Authors:** 


*A New Antagonizing Instrument for double sets of Artificial Teeth.*—Dr. James Lock, surgeon dentist of Milton, Pa., has recently sent us a very simple and at the same time quite an ingenious machine for antagonizing a full set of artificial teeth. A great variety of contrivances have been invented for this purpose; some of which have answered a tolerably good purpose, and Dr. L's, we doubt not, is as well suited to the purpose as any instruments of this description, but it is impossible to arrange a set of teeth in a machine of this sort, as accurately as in a true plaster articulating model. It is true, great care is necessary for the procurement of a correct plaster model of this sort, but when obtained, it is incomparably superior to any other means that can be resorted to, for arranging and antagonizing artificial teeth. Nevertheless, we are greatly obliged to Dr. L. for his kindness in sending us the instrument which he has invented for the purpose.—*Balt. Ed.*

